# Supplementary material for: End Sequence Analysis Toolkit (ESAT) expands the extractable information from single-cell RNA-seq data
Source: Genome Res. 2016 Oct;26(10):1397–410. doi: 10.1101/gr.207902.116 (PMC5052061; doi:10.1101/gr.207902.116)
Supplement: Supplemental Material [file supp_gr.207902.116_Supp_Materials.tar.gz › ESAT-esat_v0.1/doc/html/dir_cc8b356de703ac02f62639a9ecc046f5.html]

ESAT: java/broad/core/hmm Directory Reference


ESAT


- Main Page
- Related Pages
- Packages
- Classes
- Files

All Classes Namespaces Files Functions Variables Enumerator Pages

- java
- broad
- core
- hmm

hmm Directory Reference

Directory dependency graph for hmm:

|  |  |
| --- | --- |
| Files | |
| file | BadModelException.java |
|  | |
| file | MarkovModel.java |
|  | |
| file | MarkovState.java |
|  | |


---

Generated on Fri Aug 15 2014 15:11:15 for ESAT by  

 1.8.7
